# Alternative NHEJ pathway proteins as components of *MYCN* oncogenic activity in human neural crest stem cell differentiation: implications for neuroblastoma initiation

**DOI:** 10.1038/s41419-017-0004-9

**Published:** 2017-12-13

**Authors:** Erika A. Newman, Sahiti Chukkapalli, Daniela Bashllari, Tina T. Thomas, Raelene A. Van Noord, Elizabeth R. Lawlor, Mark J. Hoenerhoff, Anthony W. Opipari, Valerie P. Opipari

**Affiliations:** 1 Department of Surgery, C.S. Mott Children and Women’s Hospital, Mott Solid Tumor Oncology Program, The University of Michigan Medical School, Ann Arbor, MI USA; 2 Department of Pathology, The University of Michigan Medical School, Ann Arbor, MI USA; 3 Department of Pediatrics, C.S. Mott Children and Women’s Hospital, The University of Michigan Medical School, Ann Arbor, MI USA; 4 In Vivo Animal Core (IVAC), The University of Michigan Medical School, Ann Arbor, MI USA; 5 Department of Obstetrics and Gynecology, C.S. Mott Children and Women’s Hospital, The University of Michigan Medical School, Ann Arbor, MI USA

## Abstract

Neuroblastoma is a cancer of neural crest stem cell (NCSC) lineage. Signaling pathways that regulate NCSC differentiation have been implicated in neuroblastoma tumorigenesis. This is exemplified by *MYCN* oncogene targets that balance proliferation, differentiation, and cell death similarly in normal NCSC and in high-risk neuroblastoma. Our previous work discovered a survival mechanism by which *MYCN*-amplified neuroblastoma circumvents cell death by upregulating components of the error-prone non-canonical alternative nonhomologous end-joining (alt-NHEJ) DNA repair pathway. Similar to proliferating stem cells, high-risk neuroblastoma cells have enhanced DNA repair capacity, overcoming DNA damage with higher repair efficiency than somatic cells. Adequate DNA maintenance is required for lineage protection as stem cells proliferate and during tumor progression to overcome oncogene-induced replication stress. On this basis, we hypothesized that alt-NHEJ overexpression in neuroblastoma is a cancer cell survival mechanism that originates from DNA repair systems of NCSC, the presumed progenitor cell of origin. A human NCSC model was generated in which inducible *MYCN* triggered an immortalized phenotype capable of forming metastatic neuroectodermal tumors in mice, resembling human neuroblastoma. Critical alt-NHEJ components (DNA Ligase III, DNA Ligase I, and Poly [ADP-ribose polymerase 1]) were highly expressed in normal early NCSC, and decreased as cells became terminally differentiated. Constitutive *MYCN* expression maintained high alt-NHEJ protein expression, preserving the expression pattern of the immature neural phenotype. siRNA knockdown of alt-NHEJ components reversed *MYCN* effects on NCSC proliferation, invasion, and migration. DNA Ligase III, Ligase I, and PARP1 silencing significantly decreased neuroblastoma markers expression (TH, Phox2b, and TRKB). These results utilized the first human NCSC model of neuroblastoma to uncover an important link between *MYCN* and alt-NHEJ expression in developmental tumor initiation, setting precedence to investigate alt-NHEJ repair mechanics in neuroblastoma DNA maintenance.

## Introduction

Neuroblastoma (NBL), the most common extracranial tumor in children, is thought to arise from neural crest progenitor cells^1^. Signaling pathways critical for normal neural crest stem cell (NCSC) development have been implicated in NBL pathogenesis, maintaining unique embryonic properties that balance migration, proliferation, differentiation, and cell death^2^. This is highlighted by the seminal finding that targeted expression of the *MYCN onco*gene in developing neuroectodermal cells leads to NBL in transgenic mice and zebrafish models^3,4^. *MYCN* functions in early neurogenesis and is required for survival and differentiation of NCSC in distinct temporal patterns, downregulated as cells become quiescent^5–7^. Transcriptional targets of *MYCN* are also involved in many aspects of tumor biology, with dueling functions of unrestricted proliferation and cell death receptor activation^8,9^. In addition to enhanced cell growth processes, neuroblastic tumors with *MYCN* amplification develop survival mechanisms that evade death signals^10^. Evidence of this is the finding that upregulation of *MYCN* accelerates cell cycle progression and attenuates G1 checkpoint arrest^11^. In the presence of cellular stress and DNA damaging agents, an attenuated G1 checkpoint suggests that surviving NBL cells require efficient DNA maintenance pathways that circumvent apoptosis and avoid senescence^10^. This unique characteristic is distinct from somatic cells, but similar to rapidly proliferating embryonic stem cells that must maintain an effective DNA damage response despite a truncated G1 checkpoint^12^. We recently discovered a mechanism by which *MYCN*-amplified NBL circumvents DNA damage by deregulating nonhomologous end-joining (NHEJ) DNA repair components^13^. Importantly, components of alternative nonhomologous end-joining (alt-NHEJ) are upregulated in NBL cells with high-risk genetic features, while canonical nonhomologous end-joining (c-NHEJ) factors are downregulated. Compared to normal cells and non-tumorigenic NBL cell lines, high-risk lines have enhanced low-fidelity DNA repair capacity, overcoming DNA damage with significantly higher repair efficiency and high repair error rates. This survival mechanism, we hypothesize, is the result of retained NCSC-like DNA repair components that contribute to tumor maintenance.

Pluripotent stem cells have enhanced DNA repair capacity by upregulating repair pathway components for correction of damage incited by metabolic processes and collapsed replication forks^14^. The requirements for DNA double-strand break (DSB) repair during neurogenesis is selective at each stage, with spatiotemporal requirements for the two major DSB repair pathways: homologous recombination (HR) and c-NHEJ^15^. HR is the predominant DSB repair pathway during early stages of mammalian embryonic cell development, while c-NHEJ becomes the major pathway in differentiating neural progenitor cells^15,16^. NHEJ occurs in all phases of the cell cycle with higher potential for repair errors than HR, initiated by binding of Ku70/Ku80 heterodimer to broken DNA ends, and recruitment of DNA-dependent protein kinase (DNA-PKcs). c-NHEJ entails recognition and enzymatic processing at DNA break-sites to short areas of microhomology, and final ligation by XRCC4/DNA Ligase IV (Lig4)^17^.

When core c-NHEJ proteins are deficient or absent, alt-NHEJ is thought to act as a back-up nuclear DSB repair mechanism^18^. alt-NHEJ utilizes larger areas of microhomology with Poly (ADP-ribose) polymerase (PARP1) and DNA Ligase III (Lig3) or DNA Ligase 1 (Lig1) completing the critical end ligation step. Compared to c-NHEJ, alt-NHEJ components are more error-prone and have been found to drive pathogenic chromosomal translocations^19,20^. Though the precise pattern of alt-NHEJ protein expression during NCSC development has not been characterized, NHEJ is not completely eliminated when c-NHEJ components are knocked down^21^. This implies that alt-NHEJ may have a back-up role in dividing neural progenitors that is reserved for states of diminished XRCC4/Lig4 activity. Lig3, the major mediator of alt-NHEJ, is expressed at significantly higher levels in undifferentiated human embryonic stem cells compared to differentiated embryoid bodies, and may contribute to enhanced DNA repair capacity in early stem cells^22^. In this report, we specifically investigated the pattern of alt-NHEJ factors in differentiating human NCSC in order to begin to determine the role of alt-NHEJ in normal neurogenesis and in NBL tumor initiation.

To determine if alt-NHEJ component overexpression in high-risk NBL mirrors DNA repair protein expression patterns of NCSC, comparative studies of NCSC at early and late differentiation stages were performed. A novel human embryonic stem cell-derived NCSC model was generated in which inducible overexpression of *MYCN* maintained an immortalized neuroblastic phenotype. Significantly, NCSC transformed by *MYCN* (NCSC-*MYCN*) were capable of forming solid tumors in xenografted mice that histopathologically resembled NBL. This innovative model system allowed studies of protein expression of repair factors during normal neural crest differentiation and during neural crest tumor initiation. Phenotypic characterization of these tumor initiating NCSC identified an altered protein expression pattern that mirrors alt-NHEJ expression in high-risk NBL. Functional studies demonstrated that alt-NHEJ components silencing (Lig3, Lig1, PARP1) in NCSC-*MYCN* cells impaired tumorigenic properties *in vitro*. These results identify an important link between the *MYCN* oncogene and alt-NHEJ factors in early tumor initiation, and suggest that components of alt-NHEJ contribute to transformation of differentiating NCSC.

## Results

### Human NCSC have a differential NHEJ protein expression pattern

**Fig. 1:**
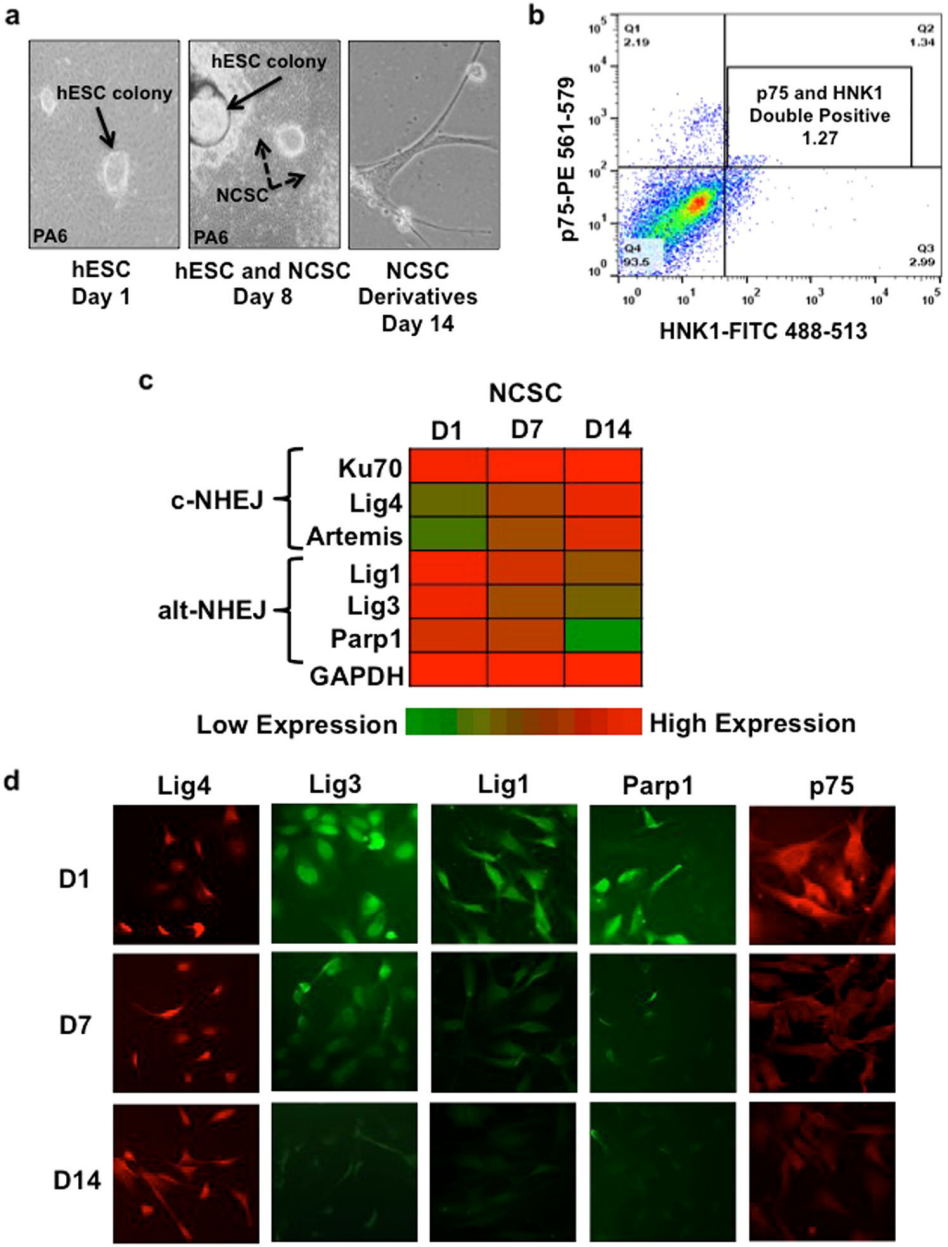
Human NCSC derived from hESC have a distinct pattern of NHEJ protein expression **a** hESC were cultured on a feeder layer of mouse embryonic fibroblasts (PA6) and placed in stromal cell-derived inducing activity (SDIA) media to promote NCSC differentiation. When cells were transitioned to neural differentiation media, there was morphologic evidence of terminal neural differentiation. Representative neural derivatives with neuronal processes are shown at day 14 in differentiation media. **b** NCSC differentiating from hESC were collected using FACS sorting. Cells were stained with p75-PE and HNK1-FITC antibodies. The double positive populations were cultured and utilized for further experiments. **c** A quantitative color map was generated from western blot analysis (shown in Supplemental Fig. S1a) of protein level expression of critical c-NHEJ (Ku70, Lig4, Artemis) and alt-NHEJ (Lig1, Lig3, PARP1) components. Protein expression was quantified using Image J software and the color plot was generated using conditional formatting in MS Excel. For each protein, the highest expression was set to a maximal of 1 (red) and lowest expression set to zero (green). The protein expression of Ku70 was consistent over all time points while Lig4 and Artemis expression increased from days 1 to 14 in differentiation media after FACS sorting, normalized for GAPDH. Significantly, all three alt-NHEJ components had high expression at Day 1 (D1) and Day 7 (D7) and were downregulated by Day 14 (D14) as NCSC differentiated in culture. **d** Representative immunocytochemistry images of NCSC expression of the c-NHEJ ligase (Lig4) and alt-NHEJ components, Lig1, Lig3, PARP1. alt-NHEJ protein expression levels decreased as p75 expression decreased with normal NCSC differentiation between time points D1 and D14

We recently discovered that components of the non-canonical alt-NHEJ repair pathway are upregulated in NBL cells with high-risk genetic features^13^. This led us to study the pattern of alt-NHEJ during differentiation of human embryonic stem cells (hESC) into NCSC, the cell of origin from which NBL arises. In order to determine the pattern of alt-NHEJ protein expression during normal NCSC differentiation, protein expression profiles of c-NHEJ and alt-NHEJ repair factors in human NCSC were examined. NCSC were generated from hESC as described by Jiang, et al. ^23^ (Fig. 1a). NCSC isolated by this method have been previously characterized as multipotent and capable of differentiation into neural crest derivatives including neurons, Schwann cells, myofibroblasts, and sympathoadrenal cells when exposed to differentiation media (see methods). hESC-derived cells were FACS-sorted after 8 days to collect cells that stained double positive for NCSC markers p75 and HNK-1 (Fig. 1b). The protein expression levels of critical c-NHEJ (Ku70, Lig4, Artemis) and alt-NHEJ factors (Lig3, Lig1, PARP1) were then assessed in these FACS-isolated cells following transfer to neural differentiation media.

The analysis revealed a distinct pattern of NHEJ repair protein expression in early NCSC compared to more differentiated neuronal precursors as detected by loss of p75 and morphologic changes consistent with neural differentiation (see right panel, Fig. 1a). Consistent with known patterns of c-NHEJ activity in neural progenitor cells^15^, Ku70 was expressed at constant levels in NCSC at all time points, while Lig4 and Artemis expression increased between early neural crest stem cells and more differentiated neural derivatives (Fig. 1c). In contrast, alt-NHEJ components Lig3, Lig1, and PARP1 were found to have high level protein expression in early NCSC that significantly decreased by day 14, with loss of p75 and morphologic differentiation (Figs. 1c and 1d). These data demonstrate that Lig3, Lig1, and PARP1 are preferentially expressed in early human NCSC and decrease as cells become terminally differentiated. This suggests that normal NCSC differentiation is associated with loss of non-canonical alt-NHEJ components. Persistent expression, therefore, may be a pathogenic factor in tumors derived from differentiating neural crest cells during development.

### Constitutive *MYCN* expression upregulates alt-NHEJ components in NCSC

**Fig. 2:**
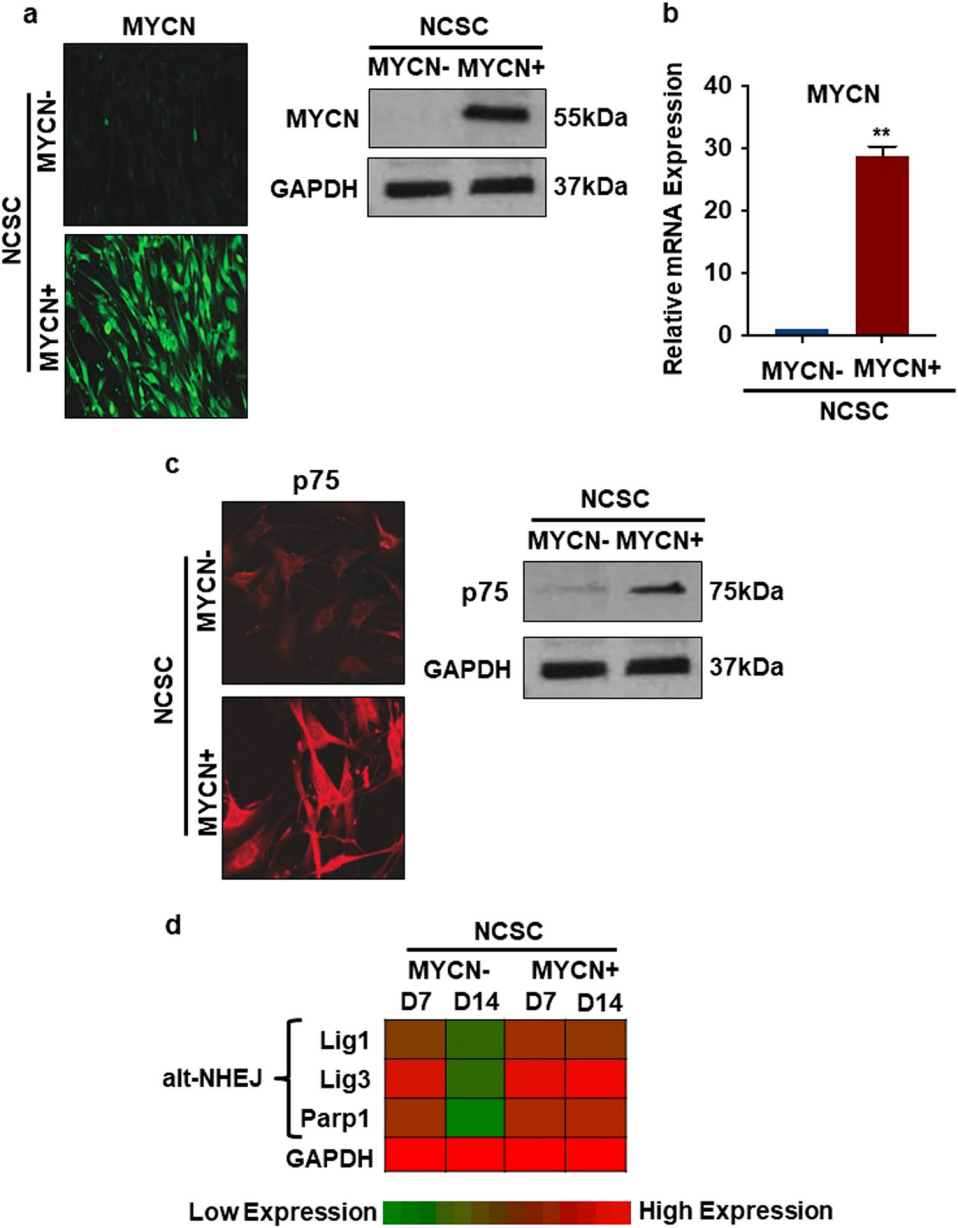
Constitutive *MYCN* expression sustains alt-NHEJ components during NCSC differentiation NCSC were double-sorted (p75, HNK-1) and plated as described. For forced *MYCN* expression, a doxycycline inducible pCL6TRRIP-*MYCN* vector was generated from a pCL6 lentiviral vector. Doxycycline was added to NCSC-*MYCN* to induce expression of *MYCN*. **a** and **b** Protein and mRNA levels of *MYCN* induction were confirmed using immunocytochemistry, western blot and RT-PCR. Representative immunocytochemistry images are shown for *MYCN-* (NCSC-MYCN without doxycycline) and *MYCN+* (NCSC-*MYCN* with doxycycline) at 24 h post *MYCN* induction. *MYCN+* cells had significant *MYCN* overexpression compared to *MYCN-* cells in all three methods. **c** Representative images of immunocytochemistry and western blot analysis for p75, an early NCSC marker are shown in *MYCN-* and *MYCN+* at D14 in differentiation media. p75 expression levels in *MYCN+* at D14 were similar to early normal NCSC at D1. **d** A quantitative color map was generated from western blot analysis (Supplemental Fig S1b) of protein level expression of alt-NHEJ components (Lig1, Lig3, PARP1). All three alt-NHEJ components tested were downregulated by D14 as normal NCSC differentiated in culture. Significantly, *MYCN+* cells maintained significantly higher expression of alt-NHEJ proteins at D7 that did not decrease by D14. Bars represent mean ± 2SD. Statistical analyses were performed with ANOVA (*n* = 3), all experiments repeated in three times, (**p < 0.01)

*MYCN*, a proto-oncogene, is the most reliable genetic marker of risk in NBL^24,25^. *MYCN* is expressed in distinct stages of neuronal development for regulation of proliferation and differentiation, and as organ systems mature, *MYCN* is turned off^7,26^. Previous work provided evidence that alt-NHEJ components function in high-risk NBL cell survival. Significantly, *MYCN* overexpression in NBL cells increases Lig3, Lig1, and PARP1 protein expression and decreases NHEJ repair accuracy^13^. Given this, we hypothesized that *MYCN* deregulation increases alt-NHEJ expression in differentiating NCSC, the NBL precursor, and that alt-NHEJ components mediate *MYCN* oncogenic activity. To study the impact of *MYCN* on the expression of alt-NHEJ components during NCSC differentiation, a doxycycline inducible vector containing the *MYCN* gene was generated (Figs. 2a and 2b). Induction of *MYCN* in early NCSC by doxycycline resulted in more than 25-fold higher *MYCN* mRNA and protein expression compared to non-transduced NCSC and NCSC-*MYCN* cells in the absence of doxycycline. *MYCN* overexpression in NCSC maintained evidence of an immature stem-like state by sustaining p75 expression at day 14 in differentiation media (Fig. 2c).

Protein expression levels of alt-NHEJ pathway components in differentiating NCSC in the presence and absence of *MYCN* were next evaluated. Compared to normally differentiating NCSC, *MYCN*-induced cells maintained high expression of all three alt-NHEJ components tested (Lig3, Lig1, PARP1), without significant changes between differentiation time points (Fig. 2d). Lig3, Lig1, and PARP1 expression remained high in NCSC with constitutive *MYCN* expression, and in contrast to normal early undifferentiated NCSC, did not significantly decrease at later time points. These results indicate that *MYCN* overexpression in NCSC maintains an immature phenotype and sustains expression of critical alt-NHEJ components, despite differentiation conditions.

### *MYCN* expression is sufficient to induce characteristics of a tumorigenic phenotype in human NCSC

**Fig. 3:**
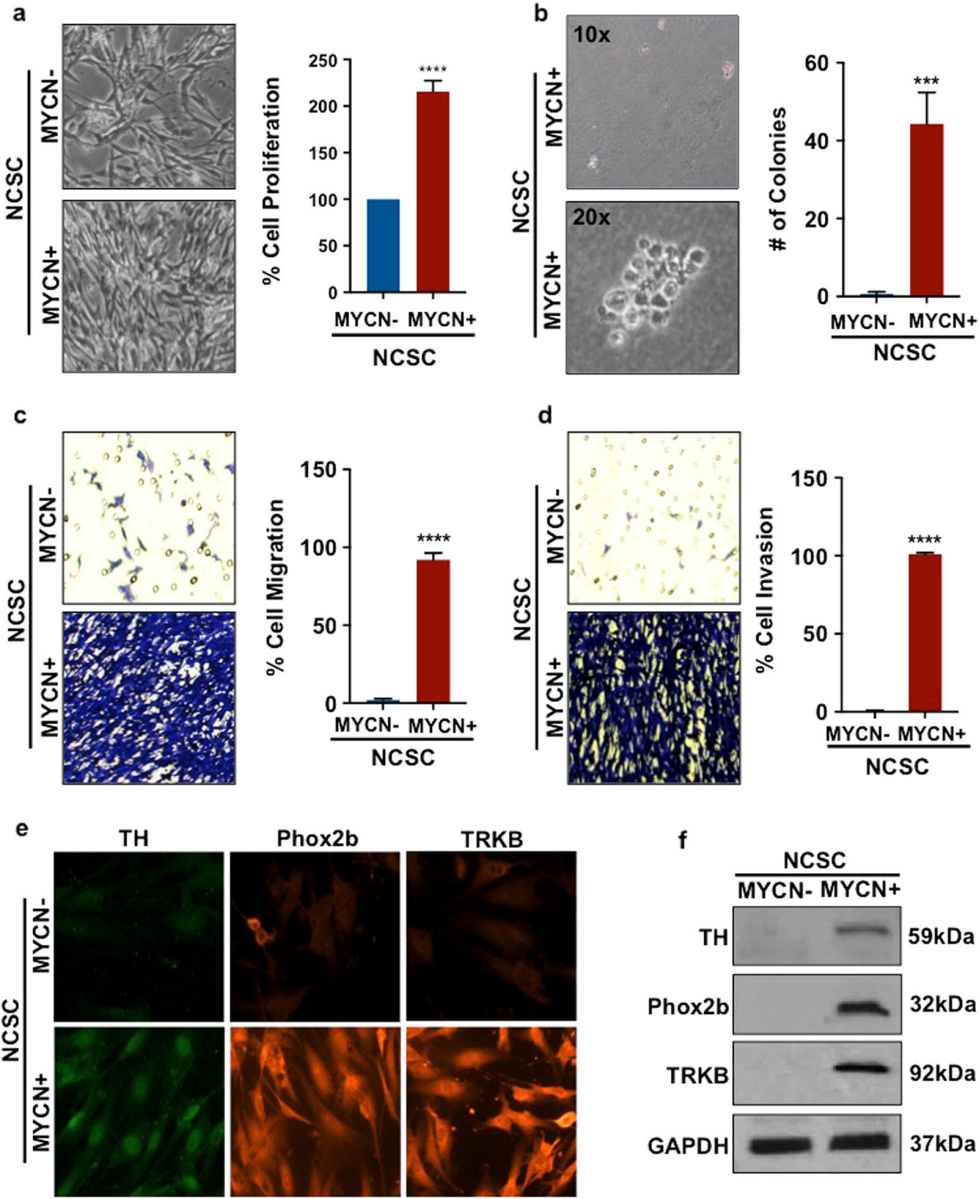
*MYCN* activation induces characteristics of a tumorigenic phenotype in differentiating NCSC **a** A MTS/PMS assay was used to measure cell proliferation. *MYCN+* cells had more than two times proliferation rates compared to *MYCN-* (****p < 0.0001). Representative microscopy images (20X) of *MYCN-* and *MYCN+* are shown. **b** Anchorage-independent growth was assessed by colony formation from single cells in soft agar.* MYCN-* and *MYCN+* were each combined in a soft agar mix and separately plated for 1 month. Soft agar plates were stained with crystal violet. Representative light microscopy images for *MYCN+* at 10× and 20× are shown. Colonies were counted manually and by ImageJ software. *MYCN+* formed over 50 colonies while *MYCN-* and controls had no colony formation. (***p < 0.001) **c:** To measure migration, *MYCN-* and *MYCN+* cells were seeded in wells of a transwell plate containing 1 µm pores. Cells that migrated through the transwell pores were stained with crystal violet and quantified. Representative light microscopy images of *MYCN-* and *MYCN+* stained with crystal violet are shown. *MYCN+* migrated significantly compared to the rate of *MYCN-* controls (****p < 0.0001). **d** Invasiveness was similarly assessed by adding a matrigel layer to the transwell porous system. *MYCN+* cells were significantly more invasive than *MYCN-* and controls (****p < 0.0001). **e** and **f** Protein expression levels of NBL tumor diagnostic markers (TH, Phox2b, and TRKB) were analyzed in *MYCN-* and *MYCN+* cells at day D14 using immunocytochemistry and immunoblotting. While *MYCN-* had minimal NBL marker expression at D14, *MYCN+* had high expression of all three NBL markers tested. Bars represent mean ± 2SD. Statistical analyses were performed with ANOVA

Disruption of *MYC* genes are known causal mutations in tumorigenesis^27^. Previous studies have shown that *MYCN* suppression inhibits NCSC growth and facilitates sympathetic neuron differentiation^26,28^. Because *MYCN* overexpression led to sustained upregulation of alt-NHEJ repair proteins in NCSC, molecular assays were conducted to further investigate phenotypic changes induced by *MYCN* in this new human NCSC-*MYCN* model. The working hypothesis was that *MYCN*-induced upregulation of alt-NHEJ components is a critical element of a broad tumorigenic state in developing NCSC. To test this, proliferation and cell viability were assessed, comparing normally developing NCSC to NCSC-*MYCN* in the presence and absence of doxycycline. Compared to normally differentiating NCSC, cells with exogenous *MYCN* expression had 2–3-fold higher proliferative rates as detected by MTS/PMS assay (Fig. 3a). To investigate the transforming potential of *MYCN* overexpression in this human NCSC model, anchorage-independent growth was assessed by colony formation from single cells in soft-agar, a measure of malignant transformation and metastatic potential^29^. Single NCSC-*MYCN* formed colonies in soft agar within 30 days, while normal NCSC and vector controls did not form colonies (Fig. 3b).

*MYCN* has also been shown to influence migration patterns, and studies in mouse and chicken embryos have demonstrated a role for *MYCN* in neural crest migratory behavior^30^. Cell migration and invasion were quantified utilizing a transwell permeable system after *MYCN* transduction. *MYCN* expression triggered migratory rates that were ten times control rates (Fig. 3c). Invasiveness was evaluated by adding matrigel to the permeable system, whereby only cells with digestive properties can traverse the transwell. *MYCN* overexpression significantly increased matrigel transwell invasion compared to NCSC controls (Fig. 3d).

Next, immunocytochemistry and western blot analysis were utilized to determine whether *MYCN* overexpression in differentiating NCSC induced neuronal markers that are diagnostic of NBL. Paired-like homeobox 2b (Phox2b), tyrosine hydroxylase (TH), and TRKB protein expression levels were analyzed in NCSC after *MYCN* transduction at 24 h (Figs. 3e and 3f). *Phox2b*, a regulator of differentiation in both normal NCSC development and in NBL tumors, was significantly increased in NCSC-*MYCN* compared to normal NCSC. Similarly, TH, a marker of sympathoadrenal cell lineage, was increased in NCSC after *MYCN* transduction. *MYCN* overexpression also induced TRKB protein expression. TRK neurotrophin receptors regulate survival and differentiation of normally developing neural precursor cells, and have been found to have a role in *MYCN*-amplified NBL clinical behavior^31^.

Together these results indicate that *MYCN* expression induces tumorigenic properties in differentiating human NCSC, modeling a malignant developmental cell phenotype that is characteristic of NBL.

### *MYCN* activation is sufficient for tumor initiation and progression in differentiating human NCSC

**Fig. 4:**
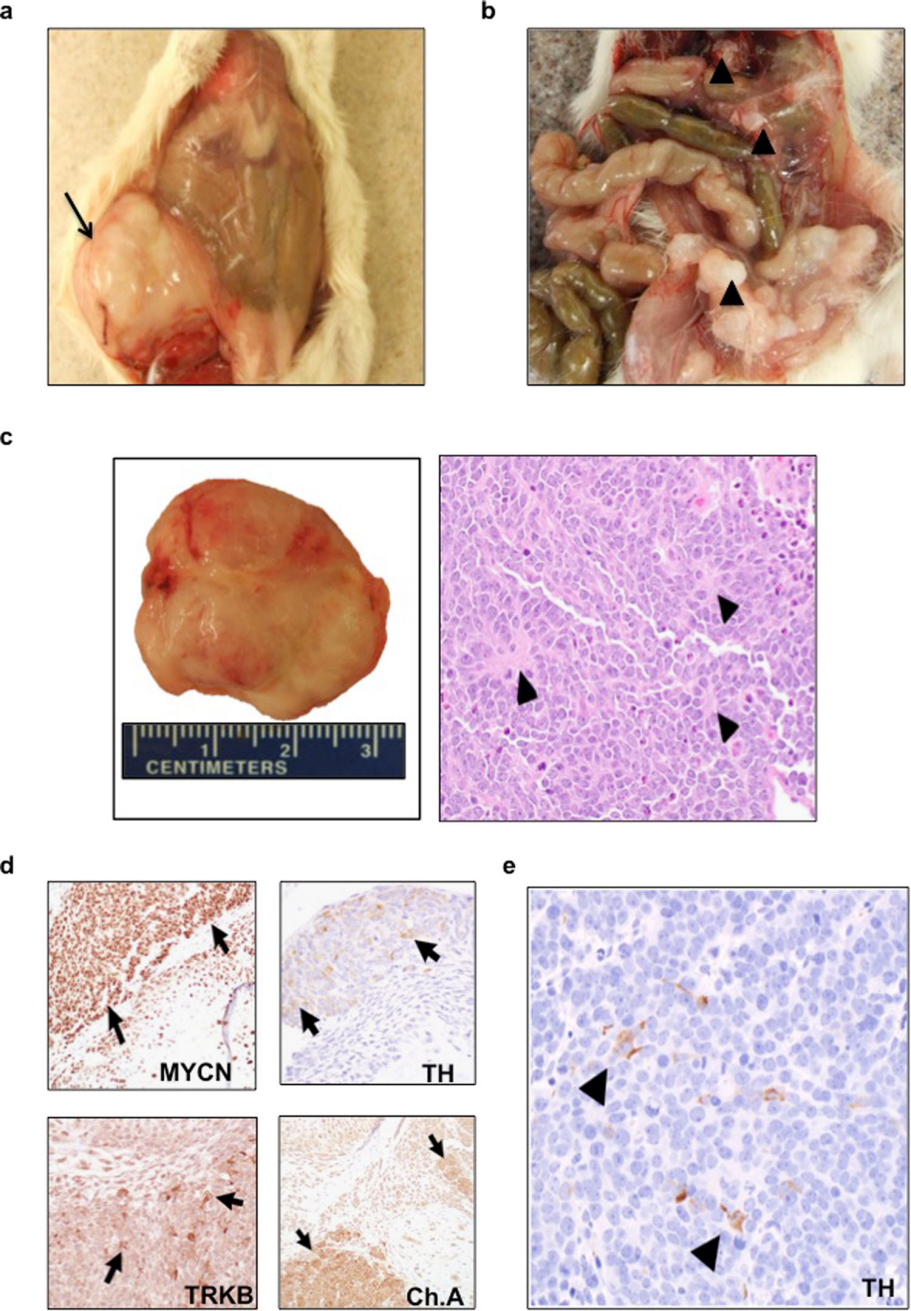
*MYCN* overexpression in human NCSC is capable of tumor initiation and progression *in vivo* NCSC transduced with *MYCN* were subcutaneously injected into the right flanks of NOD-SCID mice and normal NCSC were injected into the contralateral flanks. Mice were fed water with doxycycline for *in vivo* gene induction and monitored for local tumor formation at injection sites. **a** Large, bulky and locally invasive tumor formation occurred at NCSC-*MYCN* injection sites within 120 days with average engraftment of 67% (10/15 mice developed primary tumors). No tumors formed at the normal NCSC injection sites. **b** In addition to primary tumors at the site of injections of NCSC-*MYCN* cells, necropsy revealed evidence of distant metastatic spread in 50% of xenografts. Solid tumors metastasized to the abdominal cavity, specifically to the peritoneal surfaces and mesenteric lymph nodes. **c** Primary tumor excised and examined by H&E staining. Tumors exhibited typical primitive neuroectodermal (PNET) features with poorly differentiated basophilic cells with angular nuclei, which palisaded around blood vessels forming pseudorosettes, histologically diagnostic of human NBL. **d** NCSC-*MYCN* primary tumors were fixed and assayed by immunohistochemistry for *MYCN*, TH, TRKB, and Chromogranin A expression. *MYCN* immunolabeling was observed as distinct nuclear staining in tumor cells. Areas with primitive neuroectodermal morphology were diffusely positive for *MYCN*. Tumors with PNET morphology also displayed distinct TH, TRKB, and Chromogranin A immunolabeling in the perinuclear and cytoplasmic regions. **e** Sections of lymph node metastasis showing focal cytoplasmic immunolabeling for TH

Next, we tested whether NCSC with *MYCN* overexpression were capable of forming tumors *in vivo*. Freshly sorted NCSC-*MYCN* suspended in matrigel were injected into flanks of NOD-SCID mice, while normal NCSC controls were injected into the contralateral flanks. Animals were fed water with doxycycline for *in vivo* gene induction for the entire study period. Bilateral flanks were assessed for local tumor formation at injection sites, defined as a palpable mass attaining a volume of >300 mm^3^. Tumor engraftment and metastatic rate were the measured endpoints. Locally aggressive tumor development occurred at NCSC-*MYCN* injection sites within 120 days (average engraftment rate of 67%, average engraftment time of 114 days, 10/15 mice total), while no tumors formed at normal NCSC injection sites (Fig. 4a). Significantly, in animals that developed subcutaneous flank tumors, 50% had distant metastases to the abdominal cavity, specifically to the peritoneal surfaces and mesenteric lymph nodes (Fig. 4b, Supplemental 2). Histologically, primary tumors closely resembled human NBL, composed of poorly differentiated basophilic neuroectodermal cells with angular to elongate nuclei and scant cytoplasm that formed Homer-Wright pseudorosettes (Fig. 4c). All tumors maintained diffuse strong nuclear *MYCN* expression, and cytoplasmic labeling for markers used to characterize human NBL (TH, TRKB, and Chromogranin A) was generally focal to multifocal in small to moderate numbers of tumor cells (Fig. 4d). Cells within metastatic tumors were locally invasive and morphologically consistent with poorly differentiated NBL, with areas of focal cytoplasmic labeling for TH (Fig. 4e). These results are further evidence that *MYCN* overexpression is tumorigenic in human NCSC.

### Alt-NHEJ components are upregulated in NCSC-derived solid tumors

**Fig. 5:**
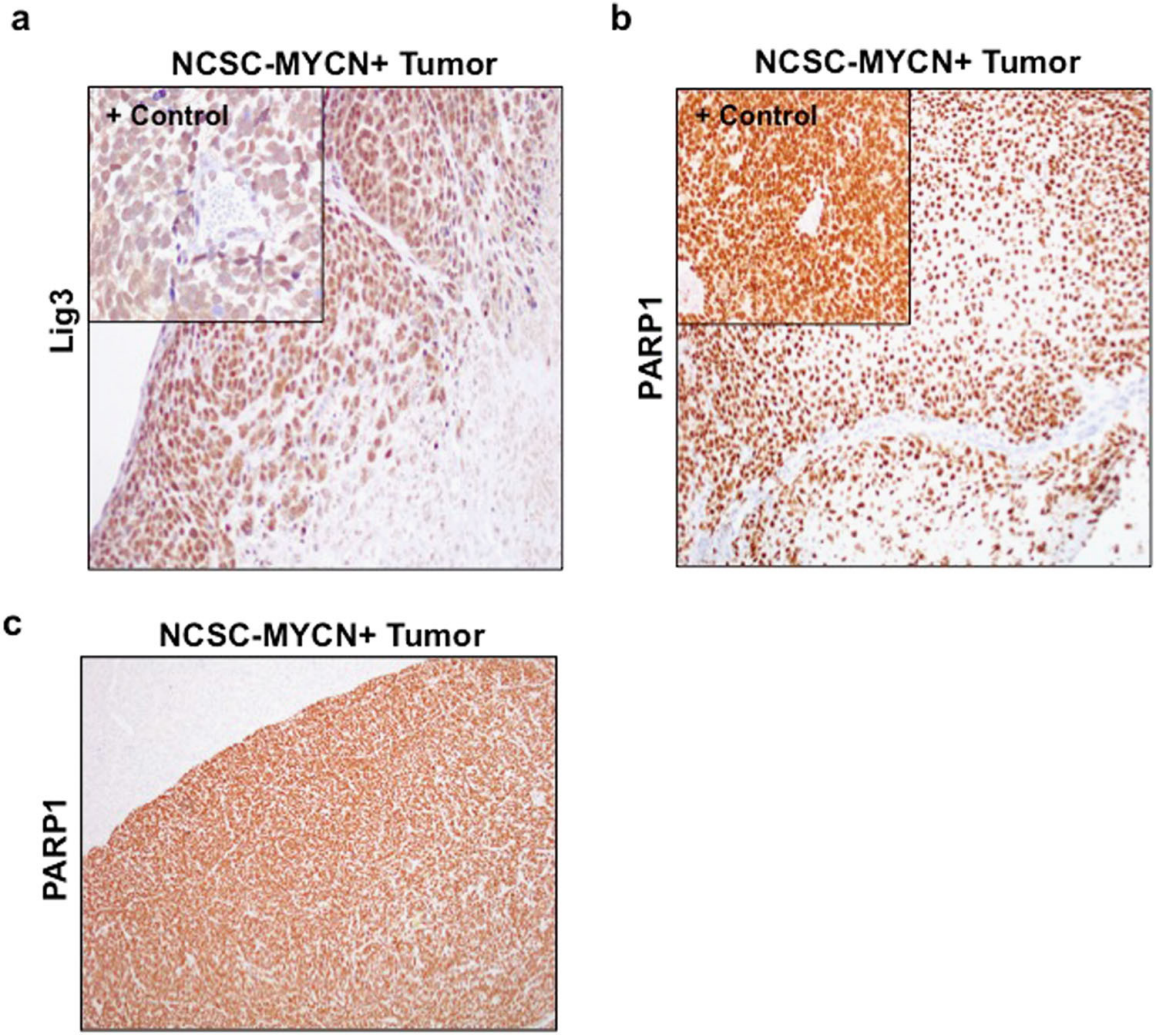
Alt-NHEJ components are upregulated in NCSC-derived solid tumors. Immunohistochemical analysis was performed on sections of tumors generated from NCSC-*MYCN* xenografts **a** Tumor specimen from IMR32 NBL xenografts (upper left panel) were utilized as positive controls for Lig3. NCSC-*MYCN* tumors had diffuse nuclear and cytoplasmic labeling in tumor sections with minimal to mild nuclear expression in adjacent tissues. **b** Positive control IMR32 NBL xenograft tumor (upper left panel) and NCSC-*MYCN* tumor with diffuse nuclear labeling of PARP1. **c** NCSC-MYCN tumor with strong and diffuse PARP1 labeling with adjacent kidney tissue deficient in PARP1 expression

*In vitro* results demonstrated that NCSC with *MYCN* overexpression resulted in upregulation of Lig3, Lig1, and PARP1. To determine whether tumors derived from NCSC maintained expression of alt-NHEJ components, immunohistochemical analysis of sections of tumors and adjoining tissues were stained for Lig3 and PARP1. NCSC-*MYCN* tumors had moderate nuclear and cytoplasmic Lig3 staining diffusely, with focal areas of nuclear labeling (Fig. 5a). In NCSC-*MYCN* tumors, all nuclei also stained strongly and diffusely positive for PARP1, while adjacent kidney tissues did not show PARP1 immunoreactivity (Figs. 5b and 5c).

### Gene silencing of alt-NHEJ components (Lig3, Lig1, and PARP1) suppresses proliferation, migration, and invasion capacities of NCSC-*MYCN*

**Fig. 6:**
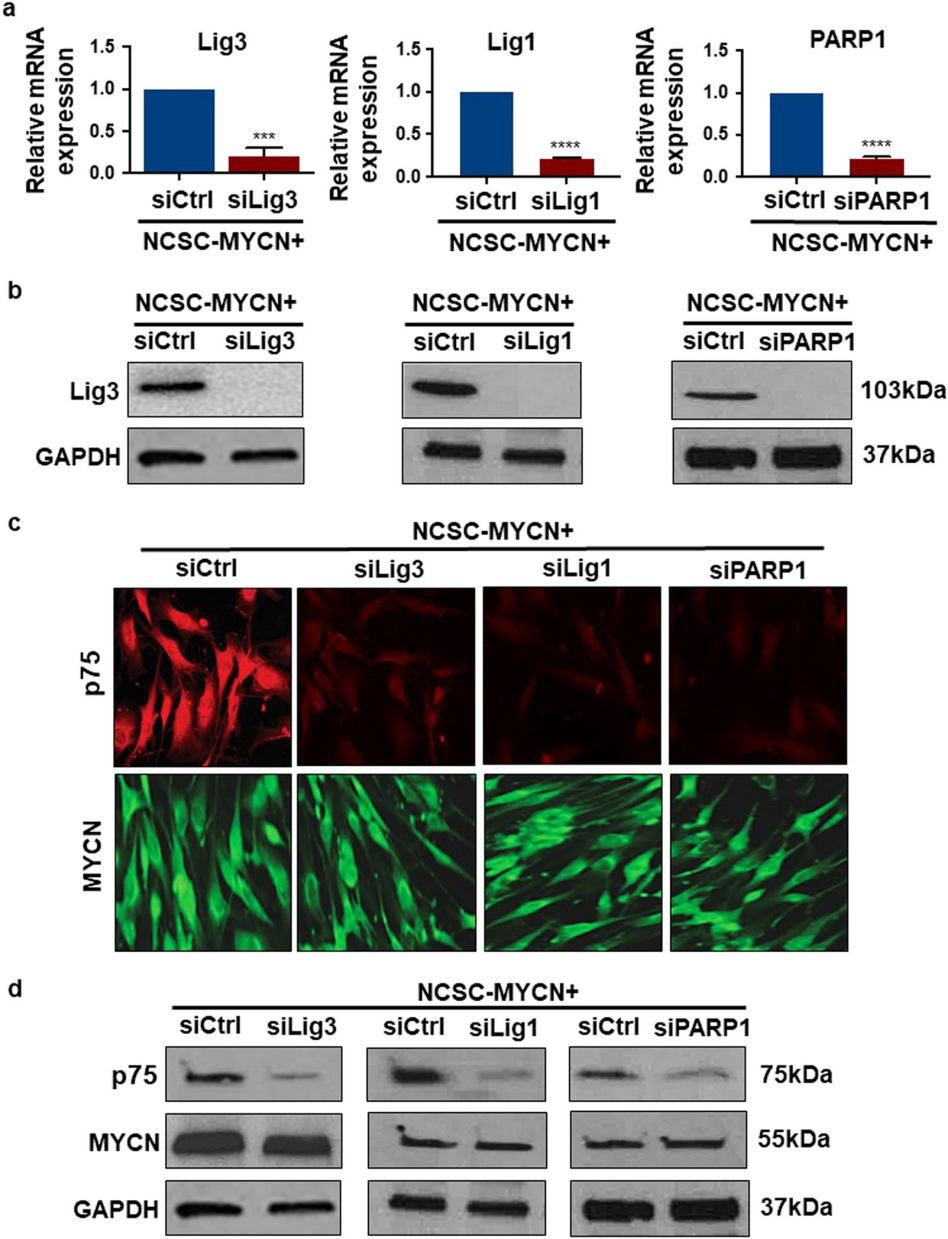
Lig3, Lig1, and PARP1 gene silencing in NCSC-*MYCN* results in loss of the NCSC-like state For genetic knockdown, NCSC-*MYCN* cells were transfected with either siLig3, siLig1, or siPARP1 with siCtrl as scrambled non-targeting control. **a** Enzymatic depletion was confirmed by qRT-PCR. NCSC-*MYCN* with siLig3, siLig1 and siPARP1 had significant decrease of respective mRNA levels compared to siCtrl. **b** Protein expression analysis of siLig3, siLig1 and siPARP1 cells also showed significant decrease in the respective enzymes. **c** and **d** Immunofluorescence and Western blot analysis for protein expression levels of *MYCN* and p75 in siLig3, siLig1, and siPARP1 showed significant decrease in expression of p75 in all three subsets compared to siCtrl. No differences were observed in *MYCN* expression patterns

High expression and maintenance of alt-NHEJ ligases coupled with *MYCN* overexpression in differentiating NCSC and in resultant tumor tissues suggests the hypothesis that alt-NHEJ components mediate *MYCN* oncogenic activity. This could be tested by inhibiting Lig3, Lig1 or PARP1 in *MYCN*-expressing NCSC and determining if this impairs the abnormal proliferation, migration and invasion capabilities of NCSC-*MYCN*. siRNA was used to specifically silence these three components individually in differentiating human NCSC-*MYCN* cells. Mock transfection and transfection with scrambled non-targeting siRNA served as controls. Relative mRNA expression of Lig3, Lig1 and PARP1 was quantified for scrambled control (siCtrl), Lig3 knockdown (siLig3), Lig1 knockdown (siLig1) and PARP1 knockdown (siPARP1) cells 72 h after transfection. Lig3, Lig1 and PARP1 expressions were significantly decreased in respective siLig3, siLig1 and siPARP1 samples when compared to controls (siCtrl) (Fig. 6a). Gene silencing was further confirmed by Western analysis that showed near complete absence of these proteins (Fig. 6b).

**Fig. 7:**
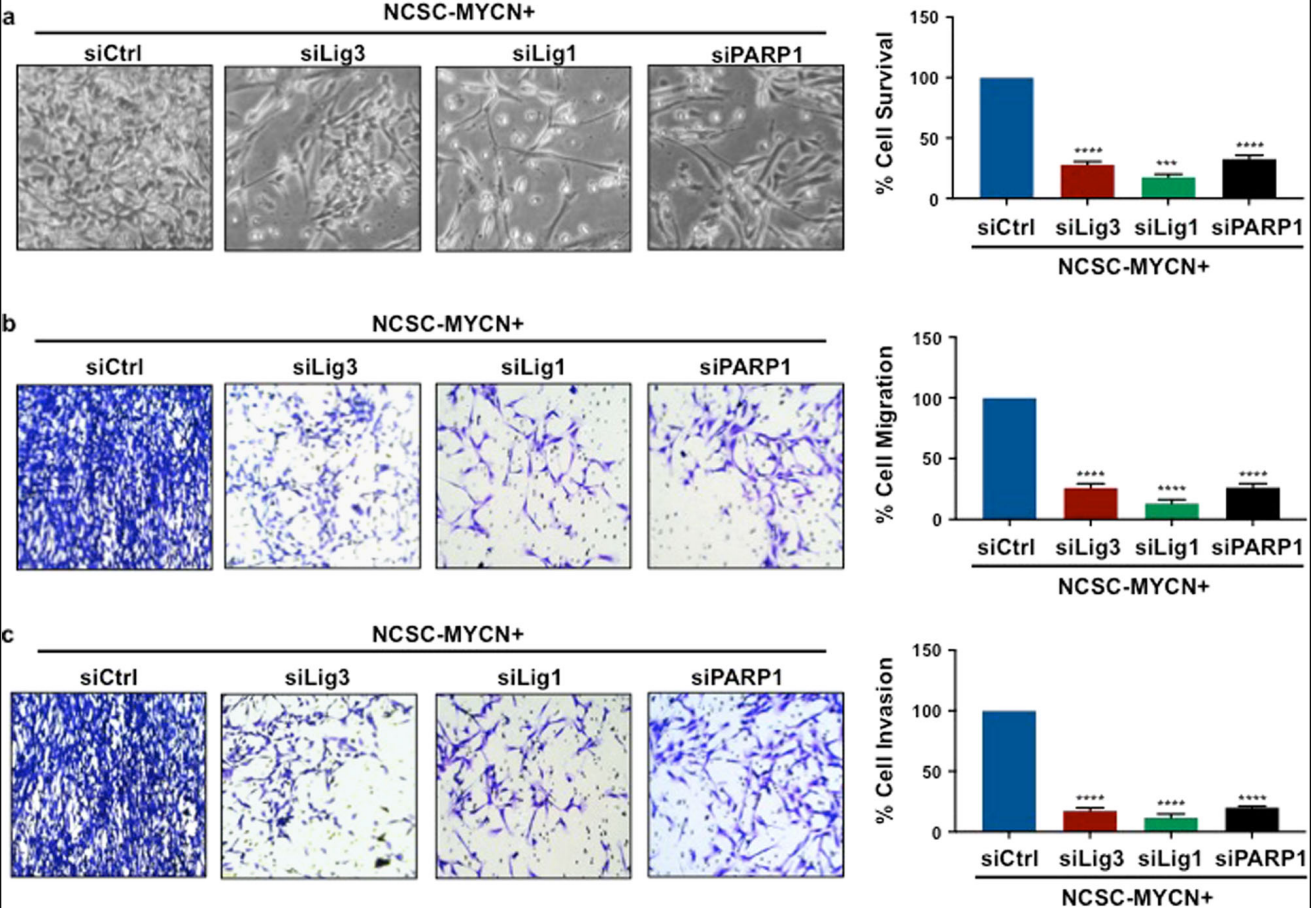
Lig3, Lig1, and PARP1 gene silencing suppresses abnormal proliferation, migration, invasion capabilities of NCSC-*MYCN* **a** A MTS/PMS assay was used to measure cell proliferation. siLig3 cells had more than 72% reduction in cell survival, siLig1 had more than 82% reduction in cell survival, and siPARP1 had more than 67% reduction in survival compared to siCtrl (***p < 0.001,****p < 0.0001). Representative microscopy images (20X) of siCtrl, siLig3, siLig1, and siPARP1 are shown. **b** and **c** All three subsets of knockdown cells had significant reduction in migration and invasion rates compared to siCtrl (****p < 0.0001). Bars represent mean ± 2SD. Statistical analyses were performed with ANOVA

**Fig. 8:**
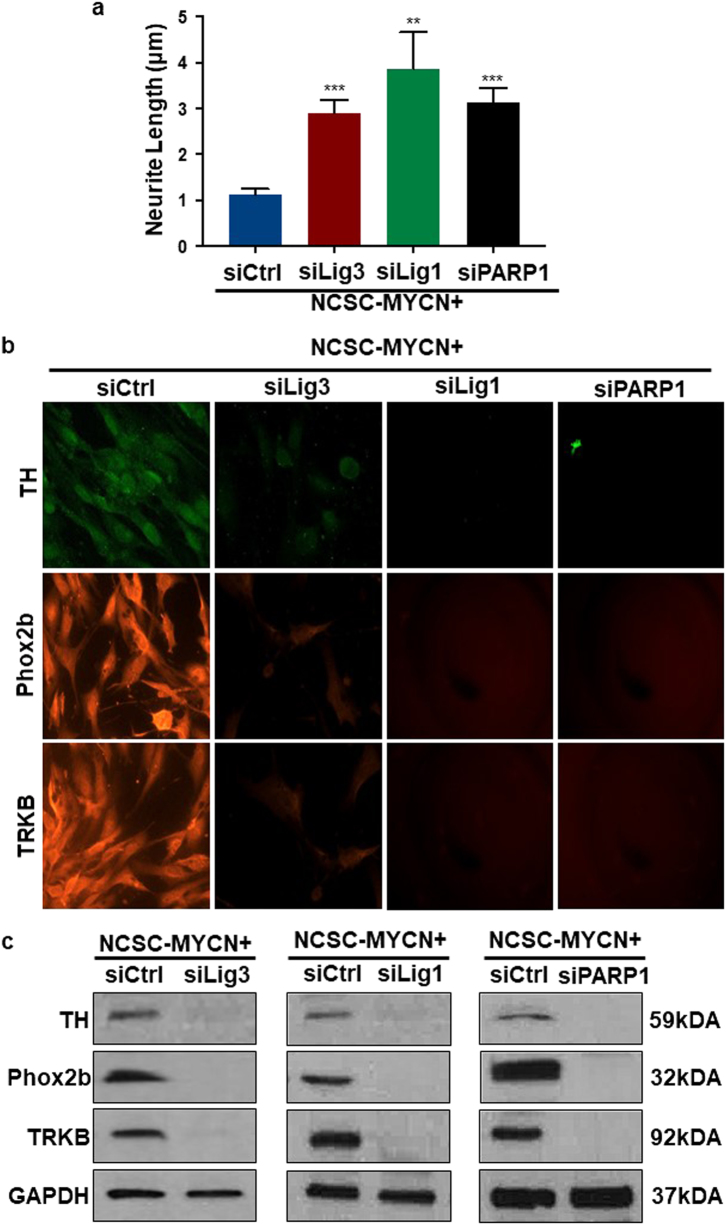
Lig3, Lig1, and PARP1 individual gene silencing in NCSC-*MYCN* cells induced a mature cell phenotype and downregulated NBL markers **a** Neurite length was measured using ImageJ. siLig3, siLig1, and siPARP1 cells had 3 to 5 times increased neurite length than compared to controls. (**p < 0.005, ***p < 0.001) **b** and **c** Protein expression levels of NBL tumor markers (TH, Phox2b, and TRKB) were determined in siCtrl, siLig3, siLig1, and siPARP1 cells using immunocytochemistry and immunoblotting. Gene silencing of alt-NHEJ components resulted in significantly decreased expression of all three NBL markers tested. Bars represent mean ± 2SD. Statistical analyses were performed with ANOVA

Lig3, Lig1 and PARP1 knockdown in NCSC-*MYCN* cells resulted in loss of immunostaining for the p75 neurotrophin receptor, indicating loss of the NCSC stem-like state (Figs. 6c and 6d). Cell survival quantification using the MTS-PMS assay demonstrated that Lig3, Lig1 and PARP1 individual knockdown were detrimental to overall NCSC-*MYCN* cell survival, decreasing survival rate by 72, 82, and 67% respectively (Fig. 7a). Cell migration and invasion assays using a transwell permeable system with crystal violet showed decrease in both cell migration and invasion by more than 50% compared to controls after Lig3, Lig1 and PARP1 individual knockdown in NCSC-*MYCN* cells (Fig. 7b and Fig. 7c). There were also distinct morphologic differences detected between alt-NHEJ components knockdown and control cells. siCtrl cells appeared more immature with spherical, densely populated cells with distinct margins, compared to knockdown cells, which appeared more elongated and neuronal-like. Cellular neurite length was significantly increased among knockdown groups compared to siCtrl, consistent with neural differentiation (Fig. 8a). Furthermore, Lig3, Lig1 and PARP1 silencing also significantly blocked protein expression of neuronal developmental and NBL tumor markers (TH, Phox2b, TRKB) in differentiating NCSC-*MYCN* cells, quantified by Western blot analysis. (Figs. 8b and 8c). Collectively, these results provide new evidence that these three factors (Lig3, Lig1, and PARP1) have a functional role in *MYC*N oncogenic activity in human NCSC, and sets precedence to investigate alt-NHEJ and non-canonical DNA repair factors in NBL tumor initiation and progression.

## Discussion

Discovery of effective therapeutics for developmental cancers such as NBL is limited by lack of reliable predictive models of tumor initiating pathways. This study provides the first hESC model of NBL derived from transformation of normally differentiating human NCSC, and further implicates neural crest progenitors as the cell-of-origin of NBL tumors. The precise time point at which neoplastic transformation occurs in differentiating NCSC remains unknown, though likely after the epithelial-mesenchymal transition (EMT), but before full sympathoadrenal differentiation^32^. This is exemplified by studies showing that tumor initiation likely occurs before the sympathoadrenal stage, as *MYC* expression in differentiated sympathoadrenal cells did not induce tumor formation in previous models^33^. It is also likely that in addition to *MYCN*, other transforming events are necessary for transformation of NCSC. Multiple signaling pathways may converge to regulate the expression of many effector genes that control growth, invasion, and migration^34^. The current results begin to define such characteristics of early p75 and HNK-1 expressing NCSC capable of tumor initiation by *MYCN* overexpression. Resultant enhanced proliferation, migratory behavior, and NBL marker expression illustrates critical association between stem cell development and tumorigenesis.

Before now, altered expression of low-fidelity DNA repair components, as a property of tumor-initiating cells has not been implicated in developmental cancers. Our initial observation that alt-NHEJ components are upregulated in early normal NCSC and decreased in more differentiated derivatives led to the question of whether there are unique expression patterns in tumorigenic progenitor cells. These findings are consistent with recent studies implicating the *MYC* family of genes in regulation of DSB repair pathways, and cells treated with MYC-specific siRNAs have reduced DNA repair activity^35^. The precise functional mechanism by which *MYCN* may drive alt-NHEJ components expression is not currently known, given the many cellular functions of *MYCN* in proliferation and replication stress. Lig3, Lig1, and PARP1 upregulation could therefore be evidence of either an enhanced DNA damage response and alt-NHEJ repair, or activated replication processes. The focus of our ongoing work is to investigate the DNA repair capacity of tumor initiating NCSC in this model to determine the specific requirement for alt-NHEJ components. To our knowledge, this has not been reported previously therefore effort is being directed towards optimizing experimental conditions to study alt-NHEJ repair metrics in neural stem cell populations. Gaining insight into the mechanistic role of Lig3 and alt-NHEJ in NBL tumor initiation and in tumorigenesis will be an important step towards implicating this pathway in NBL pathogenesis. alt-NHEJ is characterized by low-fidelity DSB repair that produces deletions and translocations, predisposing to segmental chromosomal alterations. Accumulation of segmental chromosomal alterations is linked to tumor progression in NBL and is the strongest predictor of relapse^36^. Abnormal expression of critical alt-NHEJ repair factors in NBL cells with high-risk genotypes and now in NBL progenitor cells supports a new mechanistic model in which tumor precursors may survive DNA damage by upregulation of efficient, but erroneous repair pathway components. We further reason that this may at the same time drive malignant progression by inducing segmental chromosomal alterations, an area of future investigation.

NBL progenitor cells may gain inherent DNA maintenance advantage during tumor initiation by deregulation of cancer cell-specific alt-NHEJ components, a repair process with potential for therapeutic targeting. Deregulation of DNA repair has been observed in chronic myelogenous leukemia, breast cancer, and soft tissue sarcoma, with growing evidence that NHEJ may be an important pathogenic factor in tumor maintenance^37–39^. Recent studies have identified several DSB repair lesions that may have therapeutic targeting potential^40,41^. Tumors with inherited loss of BRCA function have defects in HR DSB repair. This deficiency has been exploited by the inhibition of PARP1 in BRCA-deficient breast tumors, where spontaneous single-strand breaks result in DSB and collapsed replication forks that are left unrepaired^42^. The current results suggest that tumor initiating NCSC and derivative NBL tumors specifically upregulate alt-NHEJ proteins, components of an erroneous DNA repair pathway during periods of rapid replication. This may imply that rather than directly killing rapidly proliferating NBL cells, DNA damaging treatment strategies that don’t also target the cell’s DNA repair machinery may actually increase potential for oncogenic progression, an area for future study. In the current study, inhibition of key components of the alt-NHEJ pathway (Lig3, Lig1, PARP1) significantly impacted NCSC-*MYCN* cell survival and tumorigenic properties. An important question is whether Lig3, Lig1, or PARP1 inhibition could block tumor development, an area of our ongoing studies.

This work is therefore important because it provides first line evidence linking alt-NHEJ protein expression to NBL tumorigenesis, and sets precedence to investigate alt-NHEJ and its pathway components, which are nonessential for nuclear DNA repair in normal cells, for selective therapeutic effects in NBL.

## Materials and methods

### Cell culture

Human embryonic stem cells (hESC) (WA-H09, WiCell Research Institute, Madison, WI, USA) were cultured on irradiated mouse embryo fibroblasts (MEF) feeder layers to maintain an undifferentiated state^23^. The culture media consists of a 1:1 mixture of Dulbecco’s Modified Eagle Medium (DMEM) and nutrient mixture F-12, 20% knockout serum replacement, 1% nonessential amino acids, 1 mM l-glutamine, 4 ng/mL basic fibroblast growth culture (bFGF) (all from Invitrogen, Carlsbad, CA), and 0.1 mM 2-mercaptoethanol (Sigma Chemical Corp., St. Louis). Mouse stromal PA6 cell lines (Riken BSI Research, Japan) were cultured on gelatin-coated dishes in 90% alpha-MEM and 10% FBS (Invitrogen).

NCSC differentiation of hESC was induced according to the methods of Jiang et al, using co-culture with PA6 fibroblast for stromal-derived inducing activity^23^. Briefly, hESC colonies were treated with collagenase (Invitrogen), mechanically sectioned and transferred onto PA6-coated 60 mm dishes in neural induction (stromal derived inducing activity/SDIA) media containing Glasgow modified Eagle’s medium (GMEM), 10% knockout serum replacement, 2 mM glutamine, 1 mM pyruvate, 0.1 mM nonessential amino acid solution, and 0.1 mM 2-mercaptoethanol. On day 6, induction media was replaced and supplemented with 1X Bottenstein’s N-2 formulation (N-2 Supplement, Thermo Fisher Scientific) and replaced every 2 days. The University of Michigan Human Pluripotent Stem Cell Research Oversight committee approved all work.

### Human neural crest stem cell purification and FACS analysis

hESC were dissociated by incubating at 37 °C for 5 min in Accumax (Chemicon, Temecula, CA). Cells were pipetted and filtered through a 30μm cell strainer to collect single-cell suspensions. Cells were blocked with anti-human Fc-receptor (Miltenyi Biotec, Germany) in staining medium containing L15 medium with 10 mM HEPES and 2 mg/mL bovine serum albumin (BSA), and incubated in antibody anti-HNK-1 alone (Abcam) and anti-p75 alone (Miltenyi Biotec), at 4 °C for 20 min. HNK-1 and p75 double positive cells were isolated by FACS Aria (BD SCIENCES, San Jose, CA). Gates were defined relative to control cells and FCS Express software package (De Novo Software) was used for data analysis. NCSC that stained positive for both HNK-1 and p75 were plated on triple coated plates of 1μg/mL human laminin (Millipore, Billerica, MA), 10μg/ml poly-l-ornithine (Sigma Chemical Corp.) and 10 uμg/ml fibronectin (Gibco, Grand Island, NY). Cells were then grown in self-renewal (SR) media containing DMEM-F12 (1:1), supplemented with N2, B27, 7.5% Chick Embryo Extract (US biological, Swampscott, MA), 20 ng/mL recombinant human fibroblast growth factor-basic (bFGF), 20 ng/mL recombinant human epidermal growth factor (hEGF), 20 ng/mL insulin-like growth factor-I (IGF-I), and 0.1 mM 2-mercaptoethanol (Invitrogen). For differentiation, culture media consisted of DMEM-F12 (1:1) supplemented with N2 supplement, 200 µM ascorbic acid, and 20 ng/mL of growth factors: brain-derived neurotrophic factor (BDNF), nerve growth factor (NGF), sensory and motor neuron-derived factor (SMDF), glial cell line-derived neurotrophic factor (GDNF), and neurotrophin-3 (NT-3) (R&D systems, Minneapolis, MN).

### *MYCN* expression in human NCSC

For forced *MYCN* expression in NCSC, a pCL6TRRIP-*MYCN* vector was generated by subcloning an Age I-Nhe I *MYCN* fragment into a pCL6TRRIP inducible lentivirus vector, cut and inserted with Age I and Nhe I restriction enzymes (New England Biolabs, Ipswich, MA) upstream of a SFFV U3 promoter. To insert the *MYCN* fragment, we utilized forward 5’-CCGACCGGTCGCCACCATGCCGAGCTGCTCCAC-GTCC and the reverse 5’-CATGCTAGCCTAGCAAGTCCGAGCGTGTTC-3’ primers. The pCL6TRRIP vector was generated as previously described from the pCL6 lentiviral vector, a derivative of pCL1, and the PTRIPZ vector (Open Biosystems, Huntsville, AL)^43^. The same digestion method was used to generate a pCL6TRRIP-vector control. The resulting vectors were sequence verified at the University of Michigan Sequencing Core. All PCRs were carried out using Phusion High Fidelity Polymerase (New England Biolabs). Recombinant lentiviral particles were generated in 293T cells using a three-plasmid packing system, including our plasmid of interest, and two helper plasmids, pVSV-G and pCD/NLBH (Addgene, Cambridge, MA). Virus supernatant was collected after 48 h. Lentivirus titers of *MYCN* virus and vector controls were done following the manufacturer’s instructions using the Lentivirus qPCR titer kit from Applied Biological Materials (Richmond, BC, Canada). For transduction, human NCSC were seeded at a concentration of 1 × 10^5^ cells per well in 6 well plates in 3 mL of NCSC SR media. Cells were allowed to attach for 24 h and virus containing *MYCN* gene or vector alone was added at a multiplicity of infection ratio (MOI) of two. Cells were then treated with or without doxycycline (100 ng/mL) as appropriate for experimental conditions.

### Western blot and Immunocytochemistry

For immunocytochemistry, cells were fixed at different time points of 1, 7, and 14 days in SR Media, or 1, 7 and 14 days in differentiation media with 4% paraformaldehyde, permeabolized with 0.1% Triton X-100 for 15 min at room temperature. Slides were rinsed 3 times in 1X phosphate-buffered saline (PBS) and incubated for up to 1 h in blocking solution of 5–10% normal goat serum in PBS. Blocked slides were rinsed with PBS and incubated with primary antibodies at 1:100 overnight at 4 °C. Primary antibodies used were: *MYCN* (OP13, Millipore), Lig3 (ab587 Abcam), Lig1 (ab615 Abcam), PARP1 (sc-8007 Santa Cruz Biotechnology, Dallas, TX), Lig4 (sc-28232 Santa Cruz Biotechnology), Artemis (ab3834 Abcam), Ku70 (sc-5309 Santa Cruz Biotechnology), TH (T2928 Sigma), Phox2b (sc-376997 Santa Cruz Biotechnology), TRKB (sc-8316 Santa Cruz Biotechnology). Slides were rinsed and incubated with Alexa fluorophore-conjugated secondary antibodies for 45 min at room temperature in the dark at 1:1000 (goat anti-mouse Alexa Fluor 488, or goat-anti-rabbit Alexa Fluor 568, Thermo Fisher Scientific). Nuclei were counterstained with 4’,6-diamindino-2-phenylindole (DAPI). Stained slides were viewed using the IX83 Inverted Olympus fluorescence microscope. For immunoblotting, cell lysates were collected at different time points in RIPA lysis buffer (Santa Cruz Biotechnology) and protein concentrations were measured using bovine serum albumin as the standard with the Bradford Bio-Rad protein assay (Bio-Rad, Hercules, CA). Thirty-five micrograms of protein were resolved by SDS-Page and transferred onto a polyvinyl difluoride (PVDF) membrane (Millipore). Blocking was done with 5% milk in 1X Tris Buffered Saline with Tween-20 (TBST) for 1 h at room temperature. Primary antibodies were diluted in 5% milk and incubated overnight at 4 °C. The following antibodies were used at 1:1000 dilutions: *MYCN* (OP13, Millipore), Lig3 (ab587 Abcam), Lig1 (ab615 Abcam), PARP1 (sc-8007 Santa Cruz Biotechnology), Lig4 (sc-28232 Santa Cruz Biotechnology), Artemis (ab3834 Abcam), Ku70 (sc-5309 Santa Cruz Biotechnology), TH (T2928 Sigma), Phox2b (sc-376997 Santa Cruz Biotechnology), TRKB (sc-8316 Santa Cruz Biotechnology) and GAPDH (GT239 GeneTex, Irvine, CA). Secondary antibodies, goat anti-mouse (sc-2005, Santa Cruz Biotechnology) and goat anti-rabbit (sc-2004, Santa Cruz biotechnology), were diluted in 5% milk and incubated for 1 h at room temperature. Western Blot analyses were visualized using enhanced chemiluminescence (Amersham). Protein expression was quantified using ImageJ software (http://imagej.nih.gov/ij/). The color data plots were based on protein expression quantification, and were generated using conditional formatting in MS Excel. For each protein, the highest expression was set to a maximum of 1 (red) and the lowest expression was set to zero (green). For visual comparison, DNA repair proteins were set as fractions of the maximum.

### RNA isolation and real-time PCR

Total RNA was isolated from cells using NucleoSpin RNA kit (Machery-Nagel) according to the supplier’s instructions. The isolated RNA was then reverse transcribed using the High-Capacity cDNA Reverse Transcription Kit (Applied Biosystems). Real-time PCR was carried out on 25 ng cDNA using Fast SYBR^TM^ Green Master Mix and ViiA^TM^ 7 real-time PCR system (Applied Biosystems). All reactions were done in 20μL volume. Primers sequences for human *GAPDH* gene were: forward, 5’-AATGGGCAGCCGTTAGGAAA-3’; and reverse, 5’-GCGCCCAATACGACCAAATC-3’. Primer sequences for human *MYCN* gene were: forward, 5’-ACAGTCATCTGTCTGGACGC-3’; and reverse, 5’-TCGGAAGCAGAAAACAGTCCC-3’. Primer sequences for human *Lig3* gene were: forward, 5’-AGCTCCACTACAGGGGGTAG-3’; and reverse, 5’-GCTGGCACAGTTCTTCCTCT-3’. Primer sequences for human *Lig1* gene were: forward, 5’-CCCAGGGATTCCCCTGAAAC-3’; and reverse, 5’-GGTAGATGAGGTCGAAGGCG-3’. Primer sequences for human *PARP1* gene were: forward, 5’- GGCGATCTTGGACCGAGTAG-3’; and reverse, 5’-AGCTTCCCGAGAGTCAGGAT-3’.

### Tumorigenicity assays

Cell proliferation was measured using the MTS/PMS assay. Cells were seeded at 5 × 10^4^ density in 96 well culture plates. At end point, cells were incubated with CellTiter 96® AQueous Non-Radioactive Cell Proliferation Assay (Promega) for 4 h at 37 °C followed by absorbance read at 490 nm using a BioTek microplate reader (BioTek). To assess cell migration, transwell permeable supports with 1μm pores were used as outlined in the manufacturer’s protocol (BD Biosciences). Cells were seeded at 5 × 10^4^ density directly onto the inserts that had been triple coated with poly-l-ornithine, laminin, and fibronectin as described, and placed in BD Falcon cell culture insert companion plates (BD Biosciences). SR media was added to inserts and companion plates, and cells were allowed to migrate for 24 h. Cells that migrated to the bottom of the inserts were fixed and stained in a solution of crystal violet and methanol. Migrating cells were visualized under an inverted Olympus microscope at 40X magnification. For quantification, crystal violet stained transwell inserts were dissolved in 10% acetic acid for 15 min followed by absorbance measure at 590 nm. Invasion assessments were performed in a similar manner but BD falcon cell culture inserts were coated with matrigel matrix (BD Biosciences), blocking the pores of the inserts.

Colony formation was assessed using a soft-agar assay. Cells were cultured as described and 2500 cells per well were mixed with 0.35% noble agar (Sigma-Aldrich) in SR media, plated on top of a solidified layer of 0.5% noble agar layer in a 6 well plate. Cells were fed with fresh SR media weekly and after 3–4 weeks. The resulting colonies were dyed with 0.005% solution of crystal violet, washed with PBS, imaged using an inverted Olympus microscope, and quantified using ImageJ. Mean colony-forming efficiency was calculated as the number of colonies with >10 cells per cell inoculum x 100. For neurite measurements, bright-field images were taken of transfected groups using an inverted Olympus microscope and quantified using ImageJ software. Neurite lengths were measured for 100 cells in each image and average lengths of 100 cells and standard deviation was charted with Prism software. All experiments were repeated independently three times and results were analyzed with ANOVA. All data are presented as the mean +/- the standard deviation (SD) of three experiments, *p < 0.05, **p < 0.01, ***p < 0.001, ****p < 0.0001.

### siRNA-mediated silencing

NCSC-*MYCN* cells were plated at a density of 0.5 × 10^6^ cells per well of 6 well culture plates. On the following day, cells were transfected with either SMART-pool Lig3, SMART-pool Lig1 or SMART-pool PARP1 siRNA (Dharmacon, Inc) using a Lipofectamine RNAiMAX reagent (Invitrogen), as per the manufacturer's protocol. Transfection with scrambled siRNA served as controls. 72 h later, cells were harvested for protein and RNA. Successful depletion of Lig3, Lig1 and PARP1 was confirmed by western blot and real-time PCR. For tumorigenicity assays, cells were harvested after 72 h of transfection and seeded as described above.

### *In vivo* studies

NCSC were cultured and transduced with *MYCN* gene as described. Viable cells were identified and counted using trypan blue exclusion. For each animal injection, 2 × 10^6^ cells were re-suspended in a 1:1 ratio of PBS (Gibco) and matrigel (BD Biosciences). NOD-SCID (Charles River, Wilmington, MA) mice were subcutaneously injected with NCSC-*MYCN* cells into the right flank using 27G (BD Biosciences) iced syringes. NCSC control cells, without *MYCN* vector were injected into the left flanks. Mice were fed doxycycline water daily (2 mg/mL with 2% glucose) to induce *MYCN* gene expression. Mice were weighed, health checked, and tumor monitoring was conducted per approved IACUC protocol. Once palpable, tumors were measured with calipers, using the two greatest perpendicular tumor dimensions. Once mice met experimental endpoint, they were taken to the In Vivo Animal Core (IVAC), Unit for Laboratory Animal Medicine (ULAM) for necropsy and histopathologic evaluation by a board-certified veterinary pathologist (MJH). Animal studies were approved by the Institutional Animal Care and Usage Committee (IACUC).

### Immunohistochemistry

Paraffin blocks were sectioned at 4 um and slides were labeled with antibodies to *MYCN* (mouse monoclonal (1:100), EMD Millipore), TRKB (rabbit polyclonal (1:250), Santa Cruz Biotechnology), Chromogranin A (rabbit polyclonal (1:500), Abcam), TH (mouse monoclonal (1:1000), Sigma), Lig3 (mouse monoclonal (1:100), Abcam), and PARP1 (mouse monoclonal (1:500), Santa Cruz Biotechnology). Following antigen retrieval, quenching of endogenous peroxidase, and rodent block reagents (Biocare, Concord CA), sections were incubated with primary antibodies and washed. Following washing, mouse or rabbit polymer HRP secondary antibodies (Biocare, Concord CA) were applied. Negative controls were obtained by substitution of the primary antibody with Universal Negative reagent (Biocare, Concord CA). Following washing, 3,3-diaminobenzidine (DAB) was applied to visualize all reactions, and slides were counterstained with hematoxylin. The sections were dehydrated through graded alcohols, immersed in xylene, and mounted with coverslips.

## Electronic supplementary material

Supplemental 1

Supplemental 2
