# Supplementary figures and images for: Alternative NHEJ pathway proteins as components of MYCN oncogenic activity in human neural crest stem cell differentiation: implications for neuroblastoma initiation
Source: Cell Death Dis. 2017 Dec 13;8(12):3208. doi: 10.1038/s41419-017-0004-9 (PMC5870584; doi:10.1038/s41419-017-0004-9)

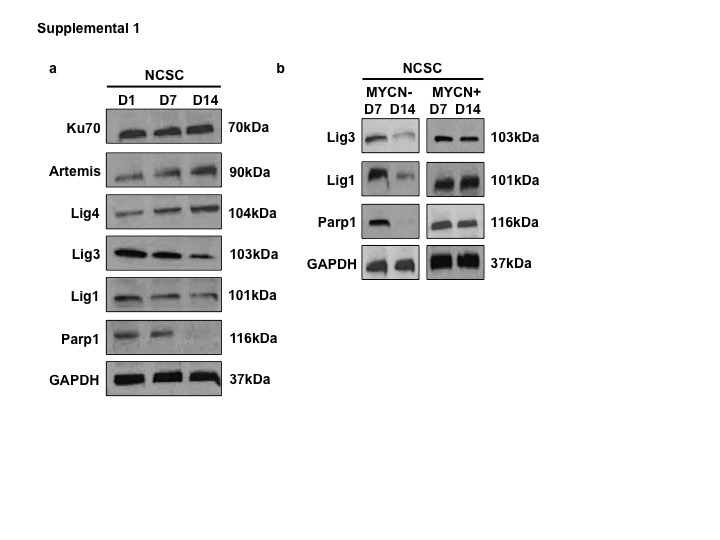

Supplement: Supplementary file 1 — Supplemental 1 [file 41419_2017_4_MOESM1_ESM.jpg]
