# Supplementary material for: Alternative NHEJ pathway proteins as components of MYCN oncogenic activity in human neural crest stem cell differentiation: implications for neuroblastoma initiation
Source: Cell Death Dis. 2017 Dec 13;8(12):3208. doi: 10.1038/s41419-017-0004-9 (PMC5870584; doi:10.1038/s41419-017-0004-9)
Supplement: Supplementary file 2 — Supplemental 2 [file 41419_2017_4_MOESM2_ESM.docx]

**Supplemental 2:**

| **Subcutaneous Engraftment** | | |
| --- | --- | --- |
|  | Primary Tumor | Metastases |
| Cell Line | Number of mice/Total mice (%) | |
| NCSC-MYCN- | 0/15 (0%) | 0/15 (0%) |
| NCSC-MYCN+ | 10/15 (67%) | 5/10 (50%) |
